# Targeting central immune signaling enhances the effects of methylphenidate in alleviating apathy-like behavior in 5xFAD mice

**DOI:** 10.21203/rs.3.rs-8031077/v1

**Published:** 2025-12-04

**Authors:** Raisa Monteiro, Jeffrey T. Dunn, Guadalupe Rodriguez, Daniel W. Fisher, Hongxin Dong

**Affiliations:** Northwestern University Feinberg School of Medicine; Northwestern University Feinberg School of Medicine; Northwestern University Feinberg School of Medicine; University of Washington School of Medicine; Northwestern University Feinberg School of Medicine

**Keywords:** Apathy, Alzheimer’s Disease, Immune System, Complement Pathway, Methylphenidate, C3ar antagonist

## Abstract

Alzheimer’s disease (AD) is frequently accompanied by neuropsychiatric symptoms (NPS), among which apathy, one of the most prevalent and burdensome, accelerates cognitive decline and disease progression, yet its molecular underpinnings remain unclear. Our previous RNA-sequencing of AD subjects revealed abnormal immune gene expression uniquely associated with apathy. In this study, we investigated whether these changes are also linked to apathy-like behavior in 5xFAD mice, and whether administration of C3a receptor antagonist SB290157, alone or with methylphenidate, modifies these behaviors. We first validated the expression of apathy-related immune hub genes identified in human AD in the prefrontal cortex (PFC) of 16-month-old 5xFAD mice using RT-qPCR. Separate cohorts of similarly aged 5xFAD and WT mice then received SB290157 and/or methylphenidate for two weeks. Results indicate that increased immune-related genes, including *Tyrobp, C3*, *C3ar*, *C1qa*, *C1qb*, and *C1qc* expression, were strongly correlated with apathy-like behavior in 5xFAD mice. Combined SB290157 and methylphenidate treatment significantly improved nest-building behavior, reduced C3 and C3ar protein expression, as well as restored dendritic spine density in the PFC. Our results confirm complement-mediated immune dysregulation is linked to apathy and suggest that co-targeting complement and catecholaminergic pathways may offer a novel therapeutic strategy for alleviating apathy in AD.

## INTRODUCTION

Alzheimer’s disease (AD) is characterized by progressive cognitive decline and eventual dementia; however, over 90% of individuals with AD also develop neuropsychiatric symptoms (NPS) during disease onset and progression ^1,2^. Apathy, defined by a lack of goal-motivated behavior ^3,4^, is one of the most prevalent NPS in AD, affecting up to 70% of individuals across all AD stages and contributing to accelerated cognitive decline, increased caregiver burden, and earlier institutionalization ^4–6^. Apathy is sometimes mistaken for depression in AD, while depression necessitates dysphoria or anhedonia, apathy is better typified as a loss of initiative, interest, or emotion in those who are otherwise euthymic ^4,7–9^. Apathy has been linked with dysfunction in several brain regions that comprise corticostriatal circuits, including the prefrontal cortex and anterior cingulate cortex ^10–12^. However, the molecular mechanisms underlying these symptoms in AD are not yet fully understood.

Although apathy is highly prevalent with a substantial negative impact on individuals with AD, there are currently no FDA-approved treatments specifically for this symptom. Existing strategies include non-pharmacological behavioral interventions and some pharmacological approaches, all of the drugs are off-label. However, a few pharmacotherapies are under investigation in clinical trials, such as methylphenidate, a psychostimulant that enhances dopaminergic and noradrenergic transmission and has shown modest improvement in apathy in multiple, randomized clinical trials ^13,14^. However, these medications come with some side effects such as adverse cardiovascular effects and weight loss that are often high-risk in a geriatric population ^15–17^. Therefore, modifying this medication and identifying new therapeutic targets for apathy in AD are sorely needed.

In a previous study, our group conducted RNA sequencing on anterior cingulate cortex (ACC) samples from postmortem AD brain tissues and correlated transcriptome patterns with ante-mortem NPS assessment 1–2 years before death ^18^. We identified numerous differentially expressed genes (DEGs) that were uniquely expressed in the ACC of individuals with AD who exhibited apathy compared to those with AD who exhibited no apathy. Weighted gene co-expression network analysis (WGCNA) identified one transcriptional module that was uniquely associated with apathy but not other NPS. This module had an overrepresentation of genes involved in central immune signaling and microglial pathways, with hub genes including *TYROBP* and complement factor C3a receptor (*C3AR*). Similar to human AD, we rigorously evaluated apathy-like behaviors in 5xFAD mice, a transgenic model of AD ^19,20^. We demonstrated that apathy-like behaviors emerge prior to the onset of memory impairment and are pervasive throughout disease progression in this model.

Therefore, to determine how the immune system hub genes might serve as targets for future pharmacotherapy in apathy, we studied these genes and their pharmacological manipulation in the 5xFAD mouse model of apathy-like behaviors. Specifically, we evaluated whether targeting the complement cascade, C3ar signaling via SB290157 ^21–23^, either alone or in combination with methylphenidate could reduce apathy-like behavior in 5xFAD mice. We hypothesized that apathy-like behavior in 5xFAD mice is facilitated by microglial phagocytic activity through complement cascade dysfunction and that antagonism of complement signaling, either alone or in combination with methylphenidate, would ameliorate this behavior.

## MATERIALS AND METHODS

### Functional enrichment analysis and network construction to validate RNA-seq findings from humans in mice.

In our prior study of the human ACC, we identified gene networks specifically linked to the apathy domain, notably the microglial phagocytic pathway and the complement cascade module ^18^. These networks encompass key immune-related pathways, including the TYROBP Causal Network (WP:WP3945), the Microglia Pathogen Phagocytosis Pathway (WP:WP3937), and the Innate Immune System pathway (REAC:R-HAS-168249). Building upon this analysis, we performed protein–protein interaction (PPI) enrichment analysis using the Search Tool for the Retrieval of Interacting Genes (STRING). Genes specifically linked to the apathy-associated module were analyzed to determine whether the resulting networks exhibited a statistically significant increase in node-to-node interactions compared to random expectation across both human and mouse genomes. A medium confidence score threshold (0.4) was applied to retain potentially biologically relevant interactions that may be underrepresented in murine datasets. Subsequent network topology analyses, including betweenness centrality, were conducted using Cytoscape (v3.10.3) to characterize key regulatory nodes within the interaction network. DEGs uniquely associated with apathy in humans were subjected to functional enrichment analysis via the Metascape platform. Annotated gene lists were exported using mouse orthologs to facilitate cross-species translational relevance for the identified human DEGs.

### Animals

Hemizygous male 5xFAD (C57BL6; APPSwFlLon, PSEN1*M146L*L286V)6799Vas/Mmjax) mice and female C57BL/6J mice were purchased from Jackson Laboratories at 6–7 weeks of age and placed for breeding in cages housing 1 male 5xFAD mouse with 1–2 female C57BL/6J mice. The litters underwent genotyping after weaning at 21 days of age by PCR with human Presenilin (*hPSEN1*) primers (Table 1). 2–5 same-sex, littermate offspring were housed in a cage with woodchip bedding and a 5 cm^2^ cotton ‘nestlet’ for enrichment. Food and water were accessible *ad libitum*, and the housing facility maintained a 12-hour light/dark cycle at a temperature of 22 ± 2°C. We divided the mice into two cohorts for this study and assessed apathy-like behaviors and memory function, as well as biochemical analyses in 5xFAD and WT mice at 16–18 months of age (168 mice in total with both sexes equally distributed). All care and use of animals took place in accordance with Northwestern University’s Institutional Animal Care and Use Committee guidelines and the NIH Guide for Care and Use of Laboratory Animals.

### Behavioral Tests

The mice were handled once per day for three consecutive days before starting the behavioral tests to reduce stress. We cleaned the test materials with 70% ethanol followed by deionized water prior to testing each animal to remove any residual scents.

### Food Burrowing

The Food Burrowing Test was carried out using slight modifications from Deacon ^24^. Burrows made from white PVC pipe (20 cm in length, 5.5 cm diameter) with one open and one closed end were used for this test. The open end was attached to glass bottles at their base (2.5 cm diameter) to raise them at a slight angle. The mice were habituated in their home cages to burrows filled with 200g of feed pellets for 24 hours. During this acclimation period, the amount burrowed was not scored, and mice remained co-housed. The next day, the mice were individually placed into new cages with filled burrows. The amount of chow burrowed was measured at two time points: 1 hour and 15 hours (overnight) after placement. Mice that removed less chow were regarded as demonstrating increased apathy-like behavior. Food burrowing behavior was evaluated twice for each animal that received the drug treatment: once before the intervention (Pre) and once after the intervention (Post).

### Nest Building

The Nest Building assay was carried out in home cages located in the animal housing facility, following the procedure designed by Deacon ^25^. Mice were placed individually into cages with a square “nestlet” of cotton bedding (5 cm^2^) at the end of each day (5 pm) for overnight testing. The next morning, nests were scored by a rater from 1 to 5 based on the quality scores developed by Deacon. The lowest nest building quality signified the highest apathy-like behavior. Half-point scores were given for nests falling between categories. The nest building test was conducted before (Pre) and after (Post) pharmacological intervention for each animal.

### Novel Object Recognition

The Novel Object Recognition (NOR) test is widely used to assess recognition memory in rodents and was implemented using a modified version of the protocol originally developed by Ennaceur and Meliani ^26,27^. For NOR test, each mouse was placed in a soundproof testing room within a Plexiglass box measuring 40 cm x 40 cm x 40 cm, evenly illuminated to maintain consistent lighting. A luxmeter was used to ensure an illumination intensity of 40 lux throughout all areas of the arena ^28^. Mouse activity was tracked using Any-Maze (Cat # 60000, Stoelting Co), object preference was scored by a researcher.

The test involves two sets of objects that are identical in height and volume but differ in shape and color. Mice were acclimated individually to the arena for 10 minutes each day for three days prior to data collection. The experimental procedure consisted of three stages: an acquisition trial lasting 10 minutes, an inter-trial interval (ITI) of one hour in their home cage, and then a retention trial also lasting 10 minutes.

During the acquisition phase, animals were observed exploring an arena that contained two identical objects placed diagonally opposite each other. After the ITI, the animals were reintroduced into the arena for the retention trial. In this trial, one of the objects was substituted with a novel object following a counterbalanced design across the cohort to avoid any side-biases in the arena. An experimenter blind to the experimental conditions recorded the time spent exploring each object during both the acquisition and retention trials from video recordings. Exploration was operationally defined as touching, leaning on, sniffing the object, or orienting the head towards the object and sniffing within a distance of less than 1.0 cm ^29^. Climbing on top of the object was not considered part of the exploration process. To remove any olfactory traces, the arena and objects were cleaned with 70% alcohol between each trial. The time spent interacting with each object during the retention trial was used to compute the discrimination index (DI), which indicates the difference in exploration time, expressed as a proportion of the total time spent examining the two objects. The discrimination index was determined using the formula:

DiscriminationIndex(DI)=(Timeexploringnovelobject-Timeexploringfamiliarobject)(Timeexploringnovelobject+Timeexploringfamiliarobject)

The NOR test was conducted only after treatment (Post) to prevent memory interference from repeated testing before and after drug treatment.

### Composite score for Apathy-like behavior

To analyze apathy-like behaviors in mice, nest building and food burrowing scores were standardized using z-score normalization ^20,30^. These z-scores were then correlated with gene expression data from the first cohort to explore the relationship between behavioral apathy and the expression of apathy-related genes. We calculated the difference of each mouse’s score from the mean score of same-aged wildtype controls. A z-score of ± 1 indicated a behavioral measurement that is ± 1 standard deviation different from the mean, always relating to the control group’s distribution. To improve reliability and reduce data variability, z-scores for nest building and 1-hour burrowing were combined into a composite apathy-like behavior score using the formula: *(z*_*i_nest*_ *+ z*_*i_burrow*_*)/2*. Overnight burrowing was excluded from the composite score because its data did not follow a normal distribution, which is required for accurate z-scoring. Additionally, overnight burrowing often shows a ceiling effect, making it less useful for detecting subtle differences between individual mice ^24^.

### Drug Treatment

The second cohort of 5xFAD and WT mice (n = 6–10/per group, total 128 mice) was used to conduct the apathy-like behavioral and memory tests to evaluate for a ‘Pre-drug condition’. After behavior and memory tests, all mice received intraperitoneal (i.p.) injections once daily for three weeks with one of the following treatments: vehicle (10% DMSO and 90% corn oil), methylphenidate (10 mg/kg; Cayman Chemical, 11639; dissolved in PBS), the C3a receptor antagonist SB290157 trifluoroacetate (1 mg/kg; MedChemExpress, HY-101502A; dissolved in 10% DMSO and 90% corn oil), or a combination of both drugs administered as separate injections on opposite sides of the abdomen. Two weeks after the drug treatment, the behavioral tests were re-conducted over the third week of treatment for the ‘Post-drug effect’ measurements.

### Euthanasia and Brain Tissue Collection

The first cohort of 5xFAD and WT mice (n = 20 per genotype, total 40) after apathy behavior testing were euthanized by pentobarbital sodium (50mg/kg i.p.) injection. Then cardiac perfusion was performed with 0.1M PBS solution for 1 minute to wash out the blood vessels in the mouse brain. The brains were collected, and the PFC was dissected from the forebrain for RT-qPCR analysis.

**Figure 1:**
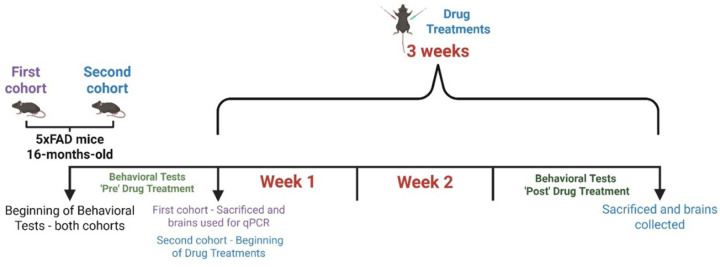
Experimental timeline schematic for animal experiments. 16-month-old 5xFAD mice were divided into two cohorts: one for qPCR analysis and another for drug treatment.

The second cohort of 5xFAD and WT mice were euthanized after completion of the ‘Post’-drug behavioral assessments and perfused in the same manner as the first cohort. Brains were then dissected and collected, and half of each brain was fixed in 4% paraformaldehyde and subsequently cryoprotected in a 30% sucrose solution for immunofluorescence. The subregions such as PFC and hippocampus of the other half of the brain were frozen for biochemical analysis. The timeline for the animal experiments is illustrated in Fig. 1.

### RT-qPCR

Frozen PFCs were lysed and processed for RNA extraction using the miRNeasy Kit (Qiagen). The qScript cDNA synthesis kit (Quantabio) was used for cDNA conversion. All primers used to test target genes are outlined in Table 1 and were designed using Primer-BLAST software and synthesized by Integrated DNA Technologies (Skokie, IL, USA). All qPCR reactions were performed using the SYBR™ Green Universal Master Mix (Applied Biosystems) on an Applied Biosystems Quantstudio 6 Flex PCR System. Fold expression change was estimated using the 2^−ΔΔ^CT method.

### Immunofluorescence

The fixed half brains were then embedded in optimal cutting temperature (OCT) medium and sectioned into 20µm thick sections through a cryostat (Leica CM1860). The coronal sections with PFC were identified and mounted on Superfrost plus slides. The sections were air dried for 10 minutes and placed for antigen retrieval in preheated citrate buffer at 95°C for 20 minutes. The sections were cooled and washed with PBS containing 0.5% Tween 20 three times for 5 minutes each on a shaker. The slides were incubated in PBS containing 0.5% Triton X-100 for permeabilization for 30 minutes after which a blocking buffer containing 1% BSA in PBS with 0.5% Triton X-100 was added for 1 hour at room temperature (RT). The slides were then placed in a humidified chamber with the primary antibody prepared in the blocking buffer and incubated at 4°C overnight. The primary antibodies and dilutions utilized for this study were C3 (1:50, sc-28294, Santa Cruz Biotech), C3ar (1:100, sc-133172, Santa Cruz Biotech), Drd1 (1:200, NBP2–16213, Novus Biologicals), and Drd2 (1:100, sc-5303, Santa Cruz Biotech). Afterward, the slides were rinsed in PBS three times, and a secondary antibody for goat anti-mouse IgG (ab97035, Abcam) or donkey anti-rabbit IgG (ab150073, Abcam) prepared in blocking buffer was added to the slides, which were incubated at RT for 1 hr. The sections were rinsed with PBS and mounted with Fluoroshield Mounting Medium with DAPI (ab104139, Abcam). The fluorescence labeled sections were imaged using a Keyence BZ-X800 microscope. The fluorescent intensity of the images was quantified using ImageJ software.

### Golgi Staining

For Golgi staining, the FD Rapid GolgiStain Kit (PK401A, FD Neuro Technologies, Inc) was used according to the manufacturer’s instructions with minor adjustments. The whole brains were collected, and olfactory bulbs removed and briefly rinsed with double distilled water (ddH2O) to remove blood. The brains were immersed in the impregnation solution consisting of equal volumes of Solutions A and B for two weeks, stored at RT in the dark. The brains were then transferred to solution C and stored at 4°C for 48 hours, after which the brains were embedded in OCT and cut into 100µm sections using a Leica CM1860 cryostat. The sections were mounted on gelatin-coated slides with a small drop of Solution C. The slides were then allowed to completely dry in the dark and used immediately. For staining, the dried slides were rinsed with ddH2O, and the sections were covered with a working solution consisting of 1 part of Solution D, 1 part of Solution E and 2 parts of ddH2O for 7 minutes. The slides were rinsed with ddH2O, and dehydrated by dipping them in 50%, 75%, and 95% ethanol. The slides were then immersed in absolute ethanol (100%), cleared with xylene, and mounted using Permount, and the images were taken using the bright-field mode of the Keyence BZ-X810 microscope. The dendritic spines from 10 different neurons over 3 different sections were quantified using ImageJ software and then averaged to get the score for every animal.

### Statistical Analysis

All statistical analyses were performed using GraphPad Prism 10. Gene expression data from male and female 5xFAD mice were compared to age-matched wild-type controls using Welch’s unpaired t-test. Correlations between apathy-like behavior and gene expression were assessed using Pearson’s correlation analysis. A three-way ANOVA was used to evaluate nest building and food burrowing behaviors across different treatment groups, genotype, and time. Two-way ANOVA was used to analyze group differences in the novel object recognition NOR test, immunohistochemistry, and Golgi staining. Tukey’s post-hoc tests were applied for multiple comparisons. Data are presented as mean ± standard error of the mean (SEM), with statistical significance defined as p < 0.05.

### Ethical approval

This article does not contain any study with human participants. All mouse experiments were carried out in accordance with the protocols described in the guidelines of the National Institutes of Health Guide for the Care and Use of Laboratory Animals and were approved by the Institutional Animal Care and Use Committee of Northwestern University (protocol number IS00009885). These studies were completed in accordance with ARRIVE guidelines.

## RESULTS

### Microglial network and the complement pathway are activated in AD individuals with apathy

**Figure 2:**
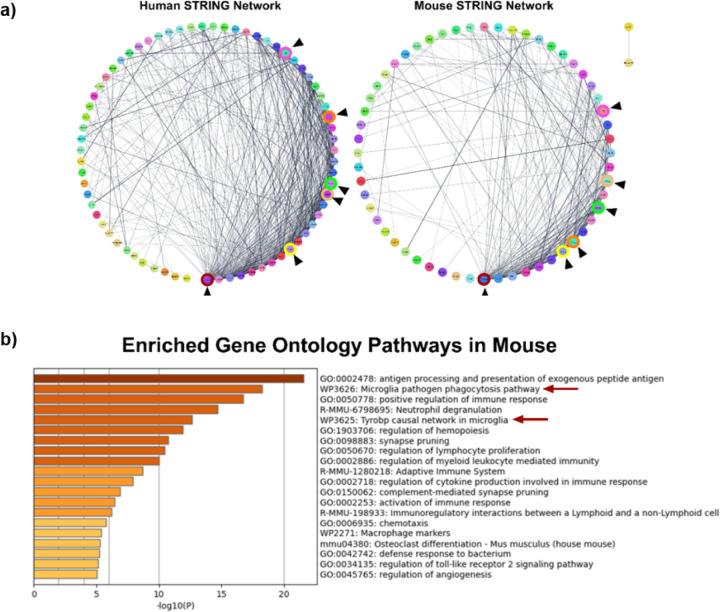
Microglial network and the complement pathway activated in apathy. (a) STRING network of the DEGs seen in the *Microglial Phagocytic Pathway and Complement Cascade* module in humans and mice. The similarity of the DEGs seen in both species indicates that the pathways seen in apathy in humans may be translational in mice. Arrowheads represent the genes seen in both humans and mice. (b) Metascape analysis of DEGs in the ‘*Microglial Phagocytic Pathway and Complement Cascade module*’, mapped from human apathy-associated profiles to mouse data, revealing repeated enrichment of the ‘Microglia pathogen phagocytosis pathway’ and the ‘Tyrobp causal network in microglia’ similar to what is observed in apathetic human AD subjects. Arrows indicate the two recurrent pathways linked to apathy in humans.

In our previous study ^18^, we found that the apathy domain in human subjects was characterized by differential expression of pathways related to the microglial complement system. Several DEGs, including *TYROBP*, *TREM2*, *CD33*, *C1QA*, *C1QB*, *C1QC*, and *C3*, were specifically associated with this domain. Comparative STRING network analysis between humans and mice revealed a set of overlapping apathy-related genes, such as *Tyrobp*, *C3*, *C1qa*, *C1qb*, *C1qc*, and *Trem2*, indicating conserved molecular signatures across species (Fig. 2A). Metascape analysis, using human data as input and mapped to mouse orthologs, identified two gene modules strongly associated with apathy: the ‘Tyrobp causal network in microglia (WP3625)’ and the ‘Microglia pathogen phagocytosis pathway (WP3626)’, as shown in Fig. 2B, which are consistent with our previous findings. The ‘Microglia pathogen phagocytosis pathway’ demonstrated high statistical significance, with Log10(P) of −18.26 and Log10(q) of −14.79. Similarly, the ‘Tyrobp causal network in microglia’ was also highly enriched, with Log10(P) of −12.65 and Log10(q) of −9.54. These results support the use of mouse models to investigate apathy-related gene dysregulation as a translational approach for mechanistic studies.

### Increased immunity gene expression in aged 5xFAD mice

**Figure 3:**
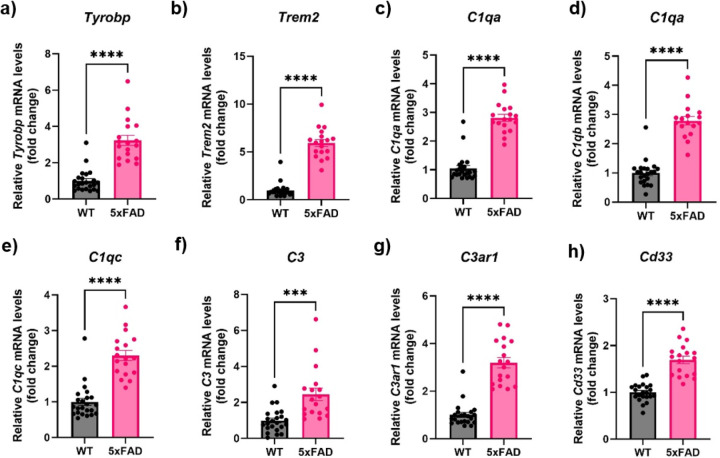
Gene expression of immune system in the PFC of 5XFAD mice. Significantly higher levels of *Tyrobp* (a), *Trem2*(b), *C1qa* (c), *C1qb* (d), *C1qc* (e), *C3* (f), *C3ar* 1 (g) and *Cd33* (h) was compared in aged 5xFAD mice (pink column) and age matched WT mice (black column) (n=12–20/group). Primers are described in Table 1. Significant differences are denoted as follows ***p<0.001 and ****p<0.0001 between WT and 5xFAD mice.

To determine how the putative apathy-related immune genes may change with AD, we performed qRT-PCR to quantify the expression of multiple immune gene in the PFC from aged (16-18-month-old) 5xFAD mice compared to wildtype (Fig. 3). The results indicated that old 5xFAD mice demonstrated a 2-fold increase in *Tyrobp* cDNA compared to WT counterparts (t(24.02) = 7.263, p < 0.0001, Fig. 3A), and a 6-fold increase for *Trem2* (t(22.7) = 12.22, p < 0.0001, Fig. 3B). The levels of the complement factor genes *C1qa* (t(34.47) = 11.22), *C1qb* (t(28.62) = 10.36) as well as *C1qc* (t(31.92) = 7.469) were also significantly higher by 2-3-fold in aged 5xFAD mice (p < 0.0001) (Fig. 3C–E). The levels of complement factor *C3* (t(22.51) = 3.899, p = 0.0007) and its receptor *C3ar1* (t(24.66) = 8.968, p < 0.0001) were significantly upregulated in 5xFAD mice as well (Fig. 3F–G). Finally, *Cd33*, another gene encoding a transmembrane protein playing an important role in immune responses, was also significantly upregulated (t(25.55) = 7.739, p < 0.0001, Fig. 3H) in aged 5xFAD mice when compared to their WT counterparts.

### Correlation of gene expression and apathy-like behavior in aged 5XFAD mice

**Figure 4:**
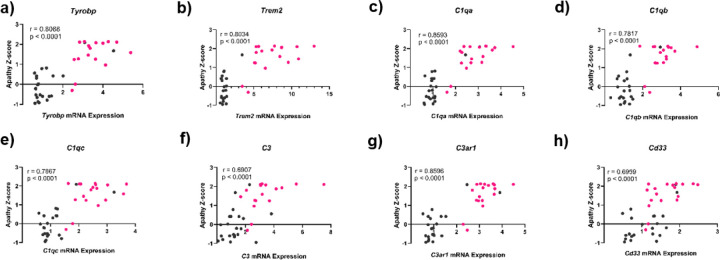
Correlation of gene expression and apathy-like behavior in 5xFAD mice. Apathy Z-score (Y-axis) for each mouse is plotted against its mRNA expression (fold change) levels (X-axis). The individual data points for WT animals (black) and 5xFAD animals (pink) are plotted on the graph for apathy and mRNA levels of *Tyrobp* (a), *Trem2* (b), *C1qa* (c), *C1qb* (d), *C1qc* (e), *C3* (f), *C3ar1* (g) and *Cd33* (h). (n=12–20/group). Significance level of correlation is denoted as p < 0.0001. ‘r’ denotes the Pearson’s correlation coefficient between the apathy z-score and mRNA expression between WT and 5xFAD mice.

To investigate the relationship between apathy-like behaviors and complement cascade gene expression, we analyzed apathy Z-scores alongside relative mRNA levels of complement-related genes in individual animals from both test groups (WT and 5xFAD) in the first cohort. Compared to WT controls, most 5xFAD mice showed elevated apathy scores and increased expression of complement cascade genes. Composite apathy scores were strongly and positively correlated with mRNA fold changes for all examined genes: *Tyrobp*, *Trem2*, *C1qa*, *C1qb*, *C1qc*, C3, *C3ar1*, and *Cd33* (p < 0.0001) (Fig. 4). Tyrobp (r = 0.8088) and Trem2 (r = 0.8034), which are upstream regulators of complement signaling, exhibited strong correlations with apathy scores (Fig. 4A–B). Genes involved in C1 signaling, including *C1qa* (r = 0.8593), *C1qb* (r = 0.7817), and *C1qc* (r = 0.7867), also showed positive associations (Fig. 4C–E). *C3* (r = 0.6907) and its receptor *C3ar1* (r = 0.8596), key components of the C3 signaling pathway, were positively correlated with apathy (Fig. 4F–G). Additionally, *Cd33* expression (r = 0.6959) was associated with apathy scores (Fig. 4H).

### Effect of pharmacological intervention on behavior and memory in aged 5xFAD mice

#### 1. Food burrowing

**Figure 5:**
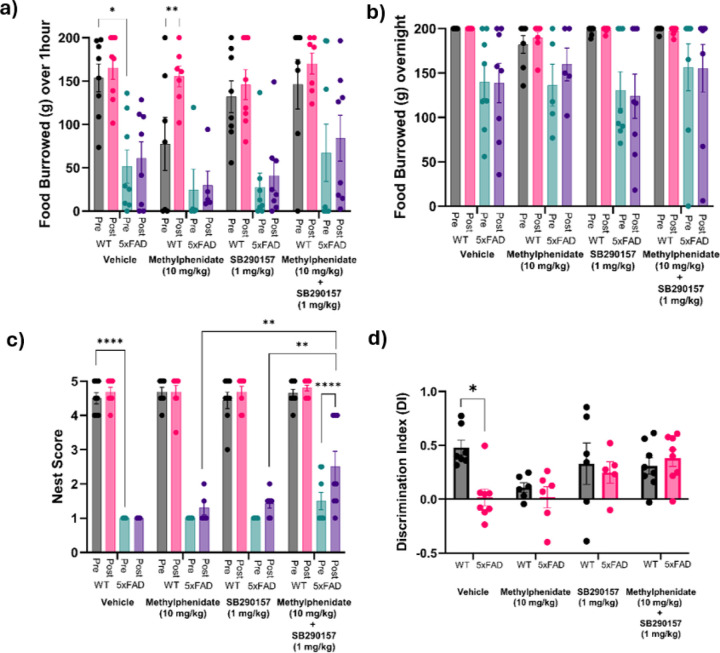
Effect of Pharmacological intervention on behavior and memory in aged 5xFAD mice. (a) Food burrowed over 1 hour by aged 5xFAD and WT controls treated with C3ar antagonist-SB290157, Methylphenidate or a combination of the two before (Pre) and after the drug regimen had been administered daily for 2 weeks (Post). (b) Food burrowed overnight by old 5xFAD and WT controls treated with C3ar antagonist-SB290157, Methylphenidate or a combination of the two before (Pre) and after the drug regimen (Post). No change was observed in food burrowing behavior in the 5xFAD animals between the different groups. (c) Nest scores of old 5xFAD and age-matched WT animals treated with C3arantagonist-SB290157, Methylphenidate or a combination of the two before (Pre) and after the pharmacological intervention (Post). Combination treatment shows improvement in nest building behavior. (d) Novel objective recognition (NOR) test for recognition memory by the discrimination index (DI) in object preference. All drug treatment groups other than the vehicle show improvement in DI in 5xFAD mice. All data expressed as mean +/− SEM. Significant differences are denoted as follows: *p<0.05,**p<0.01, **p<0.001 and ****p<0.0001 between the different treatment groups.

A three-way ANOVA was conducted to assess the amount of food burrowed by WT and 5xFAD mice before (Pre) and after (Post) treatment with vehicle, methylphenidate, SB290157, or a combination of methylphenidate and SB290157 (Fig. 5A). The 1-hour burrowing assay revealed significant main effects of genotype (F_1,51_ *=* 45.68*, p <* 0.0001) and treatment timepoint (Pre vs Post: F_1,51_ = 14.75, p = 0.0003), indicating that both genetic background and drug exposure influenced short-term burrowing behavior. However, the main effect of treatment (F_3,51_ = 1.984, p = 0.1280) and treatment × genotype (F_3,51_ = 0.1546, p = 0.9262), treatment × timepoint (F_3,51_ = 1.378, p = 0.2601), genotype × timepoint (F_1,51_ = 3.417, p = 0.0703), and treatment × genotype x timepoint interaction (F_3,51_ = 2.099, p = 0.1118) on 1-hour food burrowed were not statistically significant. Tukey’s post-hoc analysis revealed that aged 5xFAD mice treated with vehicle burrowed significantly less than WT controls (p = 0.0434). Additionally, aged WT mice receiving methylphenidate showed a significant increase in burrowing following treatment compared to baseline (5A, p=0.0012) suggesting a stimulatory effect of the drug in this cohort.

The overnight burrowing assay revealed a significant main effect of genotype (F_1,51_ = 20.30, p < 0.0001) and a significant interaction between treatment and timepoint (Pre vs Post: F_3,51_ = 3.042, p = 0.0371). All other effects, including treatment (F_3,51_ = 0.2703, p = 0.8465), timepoint (F_1,51_ = 1.287, p = 0.2620), treatment × genotype (F_3,51_ = 0.4381, p = 0.7367), genotype × timepoint (F_1,51_ = 0.1581, p = 0.6944), and the treatment × genotype x timepoint interaction (F_3,51_ = 1.081, p = 0.3655) were not significant. Tukey’s post-hoc analysis did not identify any significant differences between individual groups (Fig. 5B). Notably, there was no difference in the amount of overnight food burrowed before and after methylphenidate treatment in aged WT mice (p = 0.9984).

#### 2. Nest building scores

Three-way ANOVA revealed significant main effects of treatment (F_3,54_ = 4.893, p = 0.0044), genotype (F_1,54_ = 735.4, p < 0.0001), and timepoint (Pre vs Post: F_1,54_ = 31.88, p < 0.0001) on nest building scores. Additionally, significant interactions of treatment × genotype (F_3,54_ = 2.986, p = 0.0395), treatment × timepoint (F_3,54_ = 4.915, p = 0.0043), genotype × timepoint (F_1,54_ = 7.739, p = 0.0074), and the treatment x genotype x timepoint (F_3,54_ = 4.972, p = 0.0041) were observed on nest building scores (Fig. 5C). Tukey’s post-hoc analysis showed that 5xFAD mice exhibited significantly lower nest scores than WT mice across all treatment groups prior to intervention (p < 0.0001). Methylphenidate, with modest efficacy in human trials of apathy in AD ^14^, did not lead to statistically enhanced nest scores in 5xFAD mice after treatment (p = 0.9405) at comparable dose. Similarly, treatment with SB290157, a competitive C3ar antagonist with known anti-inflammatory and cognitive-enhancing properties ^23^, did not lead to significant changes in nest scores post-treatment at the selected dose (p = 0.2701). However, 5xFAD mice receiving the combination of methylphenidate and SB290157 showed a marked increase in nest scores following treatment (p< 0.0001 compared to Pre-treatment). Furthermore, post-hoc analysis revealed that nest scores in 5xFAD mice treated with the combination of methylphenidate and SB290157 were significantly higher compared to 5xFAD mice treated with SB290157 (p = 0.009) or methylphenidate alone (p = 0.0071). These results indicate that the combination of SB290157 and methylphenidate produces a synergistic enhancement in nest-building behavior, suggesting a more effective role for the combination of SB290157 and methylphenidate to reduce apathy-like behavior in aged 5xFAD mice.

Consistent with findings from our first cohort used for qPCR and apathy z-score analysis, the drug treatment cohort also exhibited a significant elevation in apathy z-scores in the 5xFAD group relative to WT animals prior to pharmacological intervention. Treatment with methylphenidate, SB290157, administered either individually or in combination, did not result in a statistically significant reduction in apathy z-score and were excluded from further analysis.

#### 3. Recognition memory

Our previous study indicated that 5xFAD mice at 16 months of age displayed memory deficits ^20^. In this study, we investigated whether treatment with vehicle, SB290157, methylphenidate, or a combination of methylphenidate and SB290157 could affect memory function. A two-way ANOVA revealed significant effects of genotype (F_1,46_ = 4.179, p = 0.0467), treatment (F_3,46_ = 3.114, p = 0.0352), and a genotype × treatment interaction (F_3,46_ = 3.173, p = 0.0329) on the DI. Tukey’s post-hoc analysis showed that aged 5xFAD mice treated with vehicle exhibited significantly lower DI scores compared to WT mice receiving vehicle (p = 0.0164, Fig. 5D). In contrast, DI scores with methylphenidate (p = 0.9982), SB290157 (p = 0.9994), or the combination of both drugs (p = 0.9988) were not different between 5xFAD and WT controls. These findings indicate that SB290157, methylphenidate, or their combination may alleviate memory deficits in 5xFAD mice.

### Effect of pharmacological intervention on C3 and C3ar expression in aged 5xFAD mice

**Figure 6:**
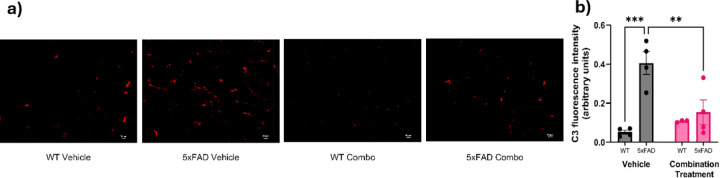
Effect of pharmacological intervention on C3expression of aged 5xFAD mice. (a) Representative staining for complement factor C3 seen across the various groups (WT-vehicle, 5xFAD-vehicle, WT-combination treatment, and 5xFAD combination treatment). The noticeable increase in red staining within the 5xFAD vehicle group suggests elevated C3levels, which decreased following the administration of the combined drug regimen. (b) Quantitative analysis of complement component C3 fluorescence intensity by ImageJ analysis (n = 3–4/group). Data are means ± SEM. Significant difference is denoted as follows: *p<0.05, **p<0.01 and ***p<0.001 between the different treatment groups.

Because the combination of SB290157 and methylphenidate may reduce apathy-like behavior in 5xFAD mice, we examined the protein levels of C3 and C3ar. Quantification of total C3 signal was done via immunohistochemistry (Fig. 6A), and two-way ANOVA revealed significant effects of genotype (F_1,11_ = 18.05, p = 0.0014) and genotype × treatment interaction (F_1,11_ = 10.65, p = 0.0075) on C3 expression (Fig. 6B). Tukey’s post-hoc analysis confirmed a significant increase in C3 fluorescence in 5xFAD-vehicle mice relative to WT-vehicle (p = 0.0002). Treatment with the combination of methylphenidate and SB290157 significantly reduced C3 fluorescence in 5xFAD mice compared to the 5xFAD-vehicle group (p = 0.0012). There was no difference in C3 levels between the WT-vehicle and WT-combination treatment groups (p = 0.4387).

**Figure 7:**
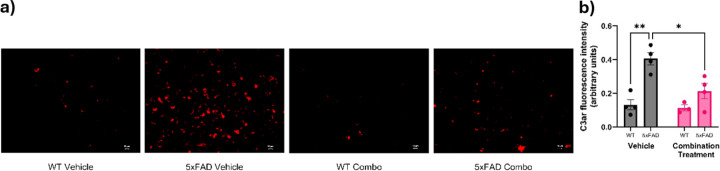
Effect of pharmacological intervention on C3arexpression of aged 5xFAD mice. (a) The representative C3ar staining in different groups (WT-vehicle, 5xFAD-vehicle, WT-combination treatment and 5xFAD combination treatment). Increased red staining in the 5xFAD vehicle group indicates increased C3arlevels, which reduced after the combined drug regimen. Scale bars, 10 μm. (b) Quantitative analysis of C3ar fluorescence intensity by ImageJ analysis (n = 3–4/group). Data are means ± SEM. Significant difference is denoted as follows: *p<0.05, **p<0.01 and ***p<0.001 between the different treatment groups.

Similarly, the intensity of C3ar (Fig. 7A) was analyzed by two-way ANOVA and revealed significant effect of genotype (F_1,11_ = 25.66, p = 0.0004, treatment (F_1,11_ = 8.202, p = 0.0154), and a genotype × treatment interaction (F_1,11_ = 5.6332, p = 0.0369) on C3ar expression (Fig. 7B). Tukey’s post-hoc analysis revealed a significant increase in C3ar in 5xFAD-vehicle animals when compared to their WT controls (p = 0.0012). Treatment of the 5xFAD animals with a combination drug led to significant reduction C3ar when compared to the 5xFAD vehicle group (p = 0.0160). The 5xFAD combination treatment group showed no significant difference from the WT-vehicle group (p = 0.5766), indicating the downregulation of C3ar levels towards normal.

### Effect of pharmacological intervention on Drd1 and Drd2 expression in aged 5xFAD mice

**Figure 8:**
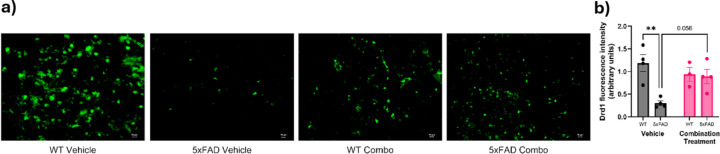
Effect of pharmacological intervention on Drd1 expression in aged 5xFAD mice. (a) Representative Drd1 staining in different groups (WT-vehicle, 5xFAD-vehicle, WT-combination treatment and 5xFAD combination treatment). Decreased green staining in the 5xFAD vehicle group indicates decreased Drd1 levels, which showed a treand towards increase after the combined drug regimen. Scale bars, 10 μm. (b) Quantitative analysis of Drd1 fluorescence intensity by ImageJ analysis. (n = 3–4/group). Data are means ± SEM. Significant difference is denoted as follows: *p<0.05, **p<0.01 and ***p<0.001 between the different treatment groups.

Methylphenidate increases extracellular dopamine by inhibiting dopamine transporters, activating Drd1 and Drd2^31^. Immunofluorescence labeling for Drd1 in the PFC of aged WT and 5xFAD animals was used to evaluate the effects of SB290157 with methylphenidate (Fig. 8). Two-way ANOVA indicated a significant effect of genotype (F_1,11_ = 10.00, p = 0.0090) and genotype × treatment interaction (F_1,11_ = 8.037, p = 0.0162) on Drd1 fluorescence (Fig. 8B). Tukey’s post-hoc test indicated a significant decrease in Drd1 in the 5xFAD-vehicle group compared to their WT controls (p = 0.0049). 5xFAD mice treated with SB290157 and methylphenidate led to a trend towards increased Drd1 compared to the 5xFAD vehicle group (p=0.056), suggesting that SB290157 and/or methylphenidate may partially restore Drd1expression in 5xFAD mice.

**Figure 9:**
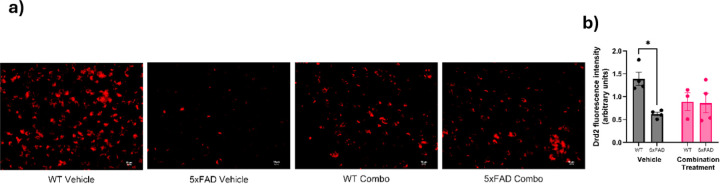
Effect of pharmacological intervention on Drd2 expression in aged 5xFAD mice. (a) Representative images of Drd2 staining in the PFC in different groups (WT-vehicle, 5xFAD-vehicle, WT-combination treatment and 5xFAD combination treatment). Decreased red foci in the 5xFAD vehicle group indicates low Drd2 levels, which showed a trend towards increase after the combined drug regimen. Scale bars, 10 μm. (b) Quantitative analysis of Drd2 fluorescence intensity by ImageJ analysis. (n = 3–4/group). Data are means ± SEM. Significant difference is denoted as follows: *p<0.05, **p<0.01 and ***p<0.001 between the different treatment groups.

Similar to Drd1, two-way ANOVA indicated a significant effect of genotype (F_1,11_ = 6.145, p = 0.0306) and genotype × treatment interaction (F_1,11_ = 5.356, p = 0.0410) on Drd2 fluorescence (Fig. 9B). Tukey’s post-hoctest indicated a significant decrease in Drd2 expression in the 5xFAD-vehicle group compared to their WT controls (p = 0.0210). Treatment with the combination drug in 5xFAD mice resulted in no significant increase in Drd2 relative to the 5xFAD-vehicle group, suggesting that combined treatment of SB290157 with methylphenidate does not affect Drd2 receptor expression.

### Effect of pharmacological intervention on dendritic spine density in aged 5xFAD micè

**Figure 10:**
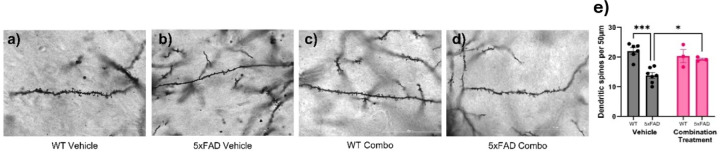
Effect of pharmacological intervention on dendritic spine density in aged 5xFAD mice. (a-d) Representative images of dendritic spines in the PFC in the different treatment groups (WT-vehicle, 5xFAD-vehicle, WT-combination treatment and 5xFAD combination treatment) taken using brightfield microscopy. WT animals dosed with vehicle showed a higher spine density which reduced significantly in the 5xFAD vehicle group.Treatment with the drug combination rescued the spinal density. Scale bars, 50 μm. (e) quantitative analysis of spines per 50 μm counted with ImageJ. (n = 3–7/group). Data are means ± SEM. Significant difference is denoted as follows: *p<0.05 and ***p<0.001 between the different treatment groups.

With Golgi staining, spines on the dendritic projections in the PFC could be clearly distinguished in the aged WT and 5xFAD mice (Fig. 10A–D). Two-way ANOVA showed a significant effect of genotype (F_1,15_ = 13.42, p = 0.0023) and genotype × treatment interaction (F_1,15_ = 7.768, p = 0.0138) on spine density. Tukey’s post-hocanalysis indicated that the decrease in spinal projections seen in 5xFAD-vehicle animals when compared to their WT controls was significant (p = 0.0002). Treatment of the 5xFAD animals with a combination of SB290157 and methylphenidate led to a significant increase in spine density when compared to the 5xFAD vehicle group (p = 0.0364), suggesting that the combination of SB290157 and Methylphenidate could restore PFC spine density in aged 5XFAD mice (Fig. 10E).

## Discussion

**Figure 11:**
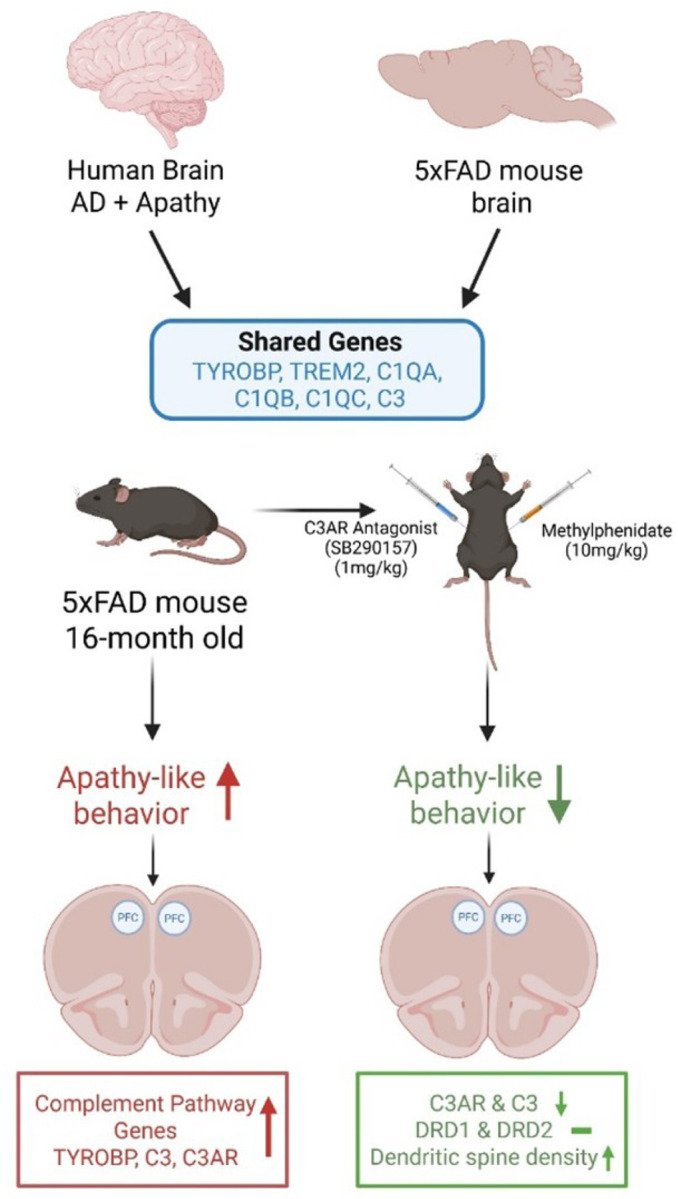
Graphical summary of the study and results. 5xFAD mice shared the dysregulated immune system genes seen in human brains with apathy and AD pathology. 5xFAD mice exhibited apathy-like behavior and combination of SB290157 with methylphenidate alleviated apathy-like behavior, reduced C3 and C3ar expression and increased the dendritic spine density in the PFC.

In this study, we found that the immune-related genes, especially those related to microglia and complement activation identified in our human RNA-seq analysis were elevated and correlated with apathy-like behaviors in 16-month-old 5xFAD mice. We then pharmacologically manipulated the C3 pathway and found that C3ar antagonist SB290157 in combination with methylphenidate, reduced apathy-like behavior as indicated by improved nest-building test scores. We speculate that these behavioral improvements may partially be due to decreased neuroinflammation, reduced complement activity, along with the restoration of dendritic spines in the PFC. Our findings further confirm our previous human work and support our hypothesis that immunity-related genes and their associated module members play a key role in regulation of apathy in AD. Fig. 11 brieflysummarizes our study and results.

In particular, consistent with our human RNA-seq findings ^18^, while differential expression of *TYROBP*, a hub gene in microglial and immune-related modules that links complement activation, synaptic pruning, and neuroinflammation, was uniquely observed in the apathy domain, we found that *Tyrobp*expression in the PFC of 16-month-old 5xFAD mice positively correlated with a composite score of apathy-like behaviors. In addition to *Tyrobp*, other candidate hub genes, including *C3*, *C1qa*, and *C1qc,* associated with complement activation, were also linked to apathy-like behaviors and were elevated in 5xFAD mice. These findings suggest that the complement cascade may contribute to apathy symptoms in AD, not only as downstream targets of *Tyrobp* but also as independent drivers of behavioral changes.

Based on our results linking increased complement activity to apathy-like behavior, we tested whether targeting C3ar, either alone or in combination with methylphenidate, could reduce apathy-like behavior in 5xFAD mice. We selected C3ar antagonist SB290157, as it is commercially available, has been tested in animal models and shown to decrease tau pathology, preserve synapses and improve behavior ^23,32,33^. However, whether it could serve as a therapeutic candidate for targeting apathy in human in AD or apathy-like behavior in an animal model has not been tested before.

Methylphenidate, a CNS stimulant, approved by the FDA for attention deficit hyperactivity disorder (ADHD), has also been investigated for the treatment of apathy in AD ^14,34,35^. Clinical trials indicate that it is generally well tolerated, though its efficacy remains modest ^14,34^. In our study, we demonstrate that at a selected dose, combining SB290157 with methylphenidate enhances its effect on apathy-like behavior in 5xFAD mice. This synergistic effect appears to act through convergent neuroinflammatory and catecholaminergic mechanisms, supporting the potential benefit of targeting both systems.

Clinical manifestations of apathy include reduced interest and motivation for daily tasks and social activities, as well as a decline in self-care ^4,36^, and these features can be modeled in animals. For example, nest building, given its strong association with self-care, has translational relevance to daily living activities in humans ^37,38^. We demonstrate that 16-month-old 5xFAD mice exhibit decreased nest-building behavior, consistent with our previous work and other studies ^20,39^. Notably, co-administration of SB290157 and methylphenidate, but not either treatment alone, significantly improved nest-building performance. In addition to nest building, we also assessed the food burrowing test, another commonly used measure of goal-directed behavior in rodents. In our study, food burrowing behavior was reduced in 5xFAD animals prior to drug treatments; however, neither drug alone nor in combination improved performance in this task. These discrepancies between the two behavioral tests suggest that nest building is a more reliable and less confounded measure of apathy in this model.

To evaluate the effect of SB290157 and/or Methylphenidate on memory function in 5xFAD mice, we conducted NOR after treatment. Vehicle-treated 5xFAD mice displayed a significantly reduced discrimination index (DI), consistent with memory impairment in this model ^40,41^. Treatment with either SB290157 or the combination with Methylphenidate, but not Methylphenidate alone, improved recognition memory. These findings support the notion that C3ar antagonism can mitigate memory deficits associated with neurodegenerative conditions ^23,33,42^.

Immunofluorescence assay showed that C3 and C3ar levels significantly increased in 5xFAD mice, in agreement with our qPCR results and prior reports ^43,44^. The reduction in C3 and C3ar expression following combination drug treatment further supports our hypothesis and highlights C3ar as a potential therapeutic target for apathy in AD. However, although both Drd1 and Drd2 levels were significantly decreased in the vehicle-treated 5xFAD mice, neither individual nor combined treatment altered dopamine receptor expression. Methylphenidate elevates extracellular dopamine levels by blocking its reuptake via the dopamine transporter. However, it does not directly alter the expression of Drd1 or Drd2, as research has shown inconsistent effects on dopamine receptor regulation ^45–49^. This disconnect indicates that increased dopamine availability may not necessarily affect receptor density, and receptor levels may remain stable or even decrease following administration of stimulants like methylphenidate due to compensatory mechanisms, including desensitization and transcriptional regulation ^47,50–52^. Our findings underscore the complexity of dopaminergic modulation in AD.

While the complement components play a critical role in synaptic pruning during brain development, dysregulation of the immune system in AD drives excessive synapse loss through microglial phagocytosis ^53,54^. Our human RNA-seq analysis revealed a strong association between apathy and the activation of microglial phagocytosis-related pathways. Overactive microglia, often referred to as disease-associated microglia (DAM), can excessively engulf neuronal structures, particularly dendritic spines, resulting in reduced spine density and contributing to neurodegeneration ^54–56^. Consistent with this, we observed a significant reduction in dendritic spine density in 5xFAD mice. Combined treatment with SB290157 and methylphenidate mitigated spine loss, potentially through suppression of microglial phagocytic activity.

Although our findings demonstrate a correlation between immune signaling pathways and the pathogenesis of apathy in AD and suggest that pharmacological targeting complement C3ar may enhance the drug efficacy of methylphenidate in managing apathy-like behavior in 5xFAD mice, a few limitations should be acknowledged. First, we did not conduct a dose-response analysis for the pharmacological treatments, and the individual contributions of SB290157 and methylphenidate to apathy-like behavior remain to be fully validated. Second, we did not directly examine microglial activation states or phagocytic activity, whether the observed spine loss in 5xFAD mice was driven by microglial-mediated phagocytosis need to be confirmed. Future research will evaluate varied dosing regimens of the individual and/or combination therapy with additional behavioral, morphological and biochemical assays to strengthen translational relevance.

## Figures and Tables

**Table 1:** Primer sequences and their gene accession numbers for PCR analysis.

Gene	Forward Primer	Reverse Primer	Gene Accession No.
*PSEN1*	GCTTTTTCCAGCTCTCATTTACTC	AAAAATGATGGAATGCTAATTGTT	NM_000021.4
*Tyrobp*	GAGGATTAAGTCCCGTACAGG	GCAGAGTCAACACCAAGTCA	NM_011662.3
*Trem2*	ACCTCTCCACCAGTTTCTCCTG	CCAGTGCTTCAAGGCGTCAT	NM_031254.4
*C1qa*	TTCCCTGAGTTTCTCTAACACC	CTGGTAAATGCGACCCTTTG	NM_007572.2
*C1qb*	GACACACCTGTTACTGCTGCTTCT	TGGTTGGCCATCAGAGCCAG	NM_009777.3
*C1qc*	TGTTCAACAGCAAGCAGGTC	TGCCCACCATGCCATTGTA	NM_007574.3
*Cd33*	GTCAGTCTCCATGAAGACTCTCC	CAGGGAACAGTCATGTTTCTGTG	NM_021293.3
*C3*	GACCAGTGAGAAGACAGTGTT	ACCACTGTCACGTACTTGTG	NM_009778.3
*C3ar1*	AGATCATCAGTCCTGGAGCCTT	CCGTGAGTGTAGGTCAGTTGAA	NM_009779.2
*Lamtor1*	ACCTTCAGCTCGCACAGAT	GTCCATGTACTCATGCTGTTCC	NM_025605.3

Experimental timeline schematic for animal experiments. 16-month-old 5xFAD mice were divided into two cohorts: one for qPCR analysis and another for drug treatment.

## Data Availability

All data that support the findings of this study are included within the article.

## References

[1] Rosenberg P. B., Nowrangi M. A. & Lyketsos C. G. Neuropsychiatric symptoms in Alzheimer’s disease: What might be associated brain circuits? Mol. Aspects Med. 43–44, 25–37. 10.1016/j.mam.2015.05.005 (2015). PMC4600424 26049034

[2] Pless A. Understanding neuropsychiatric symptoms in Alzheimer’s disease: challenges and advances in diagnosis and treatment. Front. Neurosci. 17, 1263771. 10.3389/fnins.2023.1263771 (2023). 37732300 PMC10508352

[3] Robert P. Proposed diagnostic criteria for apathy in Alzheimer’s disease and other neuropsychiatric disorders. Eur. Psychiatry. 24, 98–104. 10.1016/j.eurpsy.2008.09.001 (2009). 19201579

[4] Miller D. S. Diagnostic criteria for apathy in neurocognitive disorders. Alzheimer’s Dement. 17, 1892–1904. 10.1002/alz.12358 (2021). https://doi.org:33949763 PMC8835377

[5] Dolphin H., Dyer A. H., McHale C., O’Dowd S. & Kennelly S. P. An Update on Apathy in Alzheimer’s Disease. Geriatr. (Basel). 8. 10.3390/geriatrics8040075 (2023). PMC10366907 37489323

[6] Parrotta I. Prevalence, treatment, and neural correlates of apathy in different forms of dementia: a narrative review. Neurol. Sci. 45, 1343–1376. 10.1007/s10072-023-07197-7 (2024). 38015288 PMC10942903

[7] Teixeira A. L., Gonzales M. M., de Souza L. C. & Weisenbach S. L. Revisiting Apathy in Alzheimer’s Disease: From Conceptualization to Therapeutic Approaches. Behav Neurol 6319826 (2021). (2021). 10.1155/2021/6319826 34394772 PMC8356015

[8] Mortby M. E. Apathy as a Treatment Target in Alzheimer’s Disease: Implications for Clinical Trials. Am. J. Geriatric Psychiatry. 30, 119–147. 10.1016/j.jagp.2021.06.016 (2022). https://doi.org:. 34315645

[9] Lanctôt K. L. Distinguishing apathy from depression: A review differentiating the behavioral, neuroanatomic, and treatment-related aspects of apathy from depression in neurocognitive disorders. Int. J. Geriatr. Psychiatry. 38, e5882. 10.1002/gps.5882 (2023). 36739588 PMC10107127

[10] Levy R. & Dubois B. Apathy and the functional anatomy of the prefrontal cortex-basal ganglia circuits. Cereb. Cortex. 16, 916–928. 10.1093/cercor/bhj043 (2006). 16207933

[11] Moretti R. & Signori R. Neural Correlates for Apathy: Frontal-Prefrontal and Parietal Cortical-Subcortical Circuits. Front. Aging Neurosci. 8, 289. 10.3389/fnagi.2016.00289 (2016). 28018207 PMC5145860

[12] van Dyck C. H. Neurobiologic Rationale for Treatment of Apathy in Alzheimer’s Disease With Methylphenidate. Am. J. Geriatr. Psychiatry. 29, 51–62. 10.1016/j.jagp.2020.04.026 (2021). 32461027 PMC7641967

[13] Drye L. T. Designing a Trial to Evaluate Potential Treatments for Apathy in Dementia: The Apathy in Dementia Methylphenidate Trial (ADMET). Am. J. Geriatric Psychiatry. 21, 549–559. 10.1016/j.jagp.2012.12.018 (2013). https://doi.org:. PMC3402588 23567407

[14] Mintzer J. Effect of Methylphenidate on Apathy in Patients With Alzheimer Disease: The ADMET 2 Randomized Clinical Trial. JAMA Neurol. 78, 1324–1332. 10.1001/jamaneurol.2021.3356 (2021). 34570180 PMC8477302

[15] Garcia-Argibay M. Methylphenidate and Short-Term Cardiovascular Risk. JAMA Netw. Open. 7, e241349–e241349. 10.1001/jamanetworkopen.2024.1349 (2024). 38446477 PMC10918505

[16] Vedrenne-Gutiérrez F., S. Yu, Olivé-Madrigal A. & Fuchs-Tarlovsky V. Methylphenidate can help reduce weight, appetite, and food intake-a narrative review of adults’ anthropometric changes and feeding behaviors. Front. Nutr. 11, 1497772. 10.3389/fnut.2024.1497772 (2024). 39677498 PMC11637853

[17] Ugwendum D. Methylphenidate-Induced Non-ischemic Heart Failure With Reduced Ejection Fraction and Mild Pulmonary Hypertension. Cureus 16, e55604. 10.7759/cureus.55604 (2024). 38586757 PMC10995454

[18] Fisher D. W. Unique transcriptional signatures correlate with behavioral and psychological symptom domains in Alzheimer’s disease. Transl Psychiatry. 14, 178. 10.1038/s41398-024-02878-z (2024). 38575567 PMC10995139

[19] Locci A. Comparison of memory, affective behavior, and neuropathology in APP(NLGF) knock-in mice to 5xFAD and APP/PS1 mice. Behav. Brain Res. 404, 113192. 10.1016/j.bbr.2021.113192 (2021). 33607163 PMC7980131

[20] Keszycki R. Characterization of apathy-like behaviors in the 5xFAD mouse model of Alzheimer’s disease. Neurobiol. Aging. 126, 113–122. 10.1016/j.neurobiolaging.2023.02.012 (2023). 36989547 PMC10106415

[21] Surugiu R. Intracortical Administration of the Complement C3 Receptor Antagonist Trifluoroacetate Modulates Microglia Reaction after Brain Injury. Neural Plast 1071036 (2019). (2019). 10.1155/2019/1071036 31814819 PMC6877989

[22] Mou W., Ma L., Zhu A., Cui H. & Huang Y. Astrocyte-microglia interaction through C3/C3aR pathway modulates neuropathic pain in rats model of chronic constriction injury. Mol. Pain. 18, 17448069221140532. 10.1177/17448069221140532 (2022). 36341694 PMC9669679

[23] Yao Y. Complement C3a receptor antagonist alleviates tau pathology and ameliorates cognitive deficits in P301S mice. Brain Res. Bull. 200, 110685. 10.1016/j.brainresbull.2023.110685 (2023). https://doi.org:. 37330021

[24] Deacon R. M. J. Burrowing in rodents: a sensitive method for detecting behavioral dysfunction. Nat. Protoc. 1, 118–121. 10.1038/nprot.2006.19 (2006). 17406222

[25] Deacon R. M. J. Assessing nest building in mice. Nat. Protoc. 1, 1117–1119. 10.1038/nprot.2006.170 (2006). 17406392

[26] Ennaceur A. & Delacour J. A new one-trial test for neurobiological studies of memory in rats. 1: Behavioral data. Behav. Brain. Res. 31, 47–59. 10.1016/0166-4328(88)90157-X (1988). https://doi.org:. 3228475

[27] Ennaceur A. & Meliani K. A new one-trial test for neurobiological studies of memory in rats. III. Spatial vs. non-spatial working memory. Behav. Brain. Res. 51, 83–92. 10.1016/S0166-4328(05)80315-8 (1992). https://doi.org:. 1482548

[28] Viana T. G., Almeida-Santos A. F., Aguiar D. C. & Moreira F. A. Effects of aripiprazole, an atypical antipsychotic, on the motor alterations induced by acute ethanol administration in mice. Basic Clin. Pharmacol. Toxicol. 112, 319–324. 10.1111/bcpt.12036 (2013). 23157340

[29] Lueptow L. M. Novel Object Recognition Test for the Investigation of Learning and Memory in Mice. J. Vis. Exp. 10.3791/55718 (2017). PMC5614391 28892027

[30] Guilloux J. P., Seney M., Edgar N. & Sibille E. Integrated behavioral z-scoring increases the sensitivity and reliability of behavioral phenotyping in mice: Relevance to emotionality and sex. J. Neurosci. Methods. 197, 21–31. 10.1016/j.jneumeth.2011.01.019 (2011). 21277897 PMC3086134

[31] Volkow N. D. Methylphenidate-elicited dopamine increases in ventral striatum are associated with long-term symptom improvement in adults with attention deficit hyperactivity disorder. J. Neurosci. 32, 841–849. 10.1523/jneurosci.4461-11.2012 (2012). 22262882 PMC3350870

[32] Litvinchuk A. Complement C3aR Inactivation Attenuates Tau Pathology and Reverses an Immune Network Deregulated in Tauopathy Models and Alzheimer’s Disease. Neuron 100, 1337–1353e1335. 10.1016/j.neuron.2018.10.031 (2018). 30415998 PMC6309202

[33] Li S. A complement-microglial axis driving inhibitory synapse related protein loss might contribute to systemic inflammation-induced cognitive impairment. Int. Immunopharmacol. 87, 106814. 10.1016/j.intimp.2020.106814 (2020). https://doi.org:. 32707491

[34] Rosenberg P. B. Safety and efficacy of methylphenidate for apathy in Alzheimer’s disease: a randomized, placebo-controlled trial. J. Clin. Psychiatry. 74, 810–816. 10.4088/JCP.12m08099 (2013). 24021498 PMC3902018

[35] Verghese C., Patel P. & Abdijadid S. in StatPearls (StatPearls Publishing Copyright © 2025, StatPearls Publishing LLC., (2025).

[36] Steffens D. C., Fahed M., Manning K. J. & Wang L. The neurobiology of apathy in depression and neurocognitive impairment in older adults: a review of epidemiological, clinical, neuropsychological and biological research. Transl Psychiatry. 12, 525. 10.1038/s41398-022-02292-3 (2022). 36572691 PMC9792580

[37] Neely C. L. C., Pedemonte K. A., Boggs K. N. & Flinn J. M. Nest Building Behavior as an Early Indicator of Behavioral Deficits in Mice. JoVE e60139. 10.3791/60139 (2019). 31680688

[38] Robinson L. Apathy-like behaviour in tau mouse models of Alzheimer’s disease and frontotemporal dementia. Behav. Brain. Res. 456, 114707. 10.1016/j.bbr.2023.114707 (2024). https://doi.org:. 37820751

[39] O’Leary T. P. & Brown R. E. Age-related changes in species-typical behaviours in the 5xFAD mouse model of Alzheimer’s disease from 4 to 16 months of age. Behav. Brain Res. 465, 114970. 10.1016/j.bbr.2024.114970 (2024). 38531510

[40] Kim D. H., Kim H. A., Han Y. S., Jeon W. K. & Han J. S. Recognition memory impairments and amyloid-beta deposition of the retrosplenial cortex at the early stage of 5XFAD mice. Physiol. Behav. 222, 112891. 10.1016/j.physbeh.2020.112891 (2020). 32442584

[41] Zhu C. & Liu X. Behavioral and pathological characteristics of 5xFAD female mice in the early stage. Sci. Rep. 15, 6924. 10.1038/s41598-025-90335-2 (2025). 40011556 PMC11865263

[42] Wadhwa M. Complement activation sustains neuroinflammation and deteriorates adult neurogenesis and spatial memory impairment in rat hippocampus following sleep deprivation. Brain Behav. Immun. 82, 129–144. 10.1016/j.bbi.2019.08.004 (2019). 31408672

[43] Ruiz-Pérez G. Potentiation of amyloid beta phagocytosis and amelioration of synaptic dysfunction upon FAAH deletion in a mouse model of Alzheimer’s disease. J. Neuroinflamm. 18, 223. 10.1186/s12974-021-02276-y (2021). PMC8482614 34587978

[44] Nakano-Kobayashi A., Canela A., Yoshihara T. & Hagiwara M. Astrocyte-targeting therapy rescues cognitive impairment caused by neuroinflammation via the Nrf2 pathway. Proceedings of the National Academy of Sciences 120, e2303809120 (2023). 10.1073/pnas.2303809120 PMC10438385 37549281

[45] Papa M., Sellitti S. & Sadile A. G. Remodeling of neural networks in the anterior forebrain of an animal model of hyperactivity and attention deficits as monitored by molecular imaging probes. Neurosci. Biobehavioral Reviews. 24, 149–156. 10.1016/S0149-7634(99)00052-4 (2000). 10654672

[46] Marco E. M. Neurobehavioral adaptations to methylphenidate: The issue of early adolescent exposure. Neurosci. Biobehavioral Reviews. 35, 1722–1739. 10.1016/j.neubiorev.2011.02.011 (2011). https://doi.org:. 21376076

[47] Caprioli D. Dissociable rate-dependent effects of oral methylphenidate on impulsivity and D2/3 receptor availability in the striatum. J. Neurosci. 35, 3747–3755. 10.1523/jneurosci.3890-14.2015 (2015). 25740505 PMC4348181

[48] Stevens T., Sangkuhl K., Brown J. T., Altman R. B. & Klein T. E. PharmGKB summary: methylphenidate pathway, pharmacokinetics/pharmacodynamics. Pharmacogenet Genomics. 29, 136–154. 10.1097/fpc.0000000000000376 (2019). 30950912 PMC6581573

[49] Klein S. R., Blum K., Gold M. S. & Thanos P. K. Chronic Methylphenidate Effects on Brain Gene Expression: An Exploratory Review. Psychol. Res. Behav. Manag. 17, 577–592. 10.2147/prbm.S445719 (2024). 38379637 PMC10876479

[50] Sibley D. R. & Neve K. A. in in The Dopamine Receptors. 383–424 (eds Neve K. A., Rachael L.& Neve) (Humana, 1997).

[51] Ashok A. H., Mizuno Y., Volkow N. D. & Howes O. D. Association of Stimulant Use With Dopaminergic Alterations in Users of Cocaine, Amphetamine, or Methamphetamine: A Systematic Review and Meta-analysis. JAMA Psychiatry. 74, 511–519. 10.1001/jamapsychiatry.2017.0135 (2017). 28297025 PMC5419581

[52] Zetterström T. S. C., Quansah E. & Grootveld M. in New Discoveries in the Behavioral Neuroscience of Attention-Deficit Hyperactivity Disorder (eds Clare Stanford S. & Sciberras Emma) 127–157 Springer International Publishing, (2022).

[53] Hong S., Dissing-Olesen L. & Stevens B. New insights on the role of microglia in synaptic pruning in health and disease. Curr. Opin. Neurobiol. 36, 128–134. 10.1016/j.conb.2015.12.004 (2016). 26745839 PMC5479435

[54] Carpanini S. M. Terminal complement pathway activation drives synaptic loss in Alzheimer’s disease models. Acta Neuropathol. Commun. 10, 99. 10.1186/s40478-022-01404-w (2022). 35794654 PMC9258209

[55] Fricker M., Oliva-Martín M. J. & Brown G. C. Primary phagocytosis of viable neurons by microglia activated with LPS or Aβ is dependent on calreticulin/LRP phagocytic signalling. J. Neuroinflamm. 9, 196. 10.1186/1742-2094-9-196 (2012). PMC3481398 22889139

[56] Cheng J. Microglial Calhm2 regulates neuroinflammation and contributes to Alzheimer’s disease pathology. Sci. Adv. 7 10.1126/sciadv.abe3600 (2021). PMC8386937 34433553

